# We need to embed implementation research in immunization programmes in Africa

**DOI:** 10.11604/pamj.supp.2025.51.1.46841

**Published:** 2025-06-13

**Authors:** Charles Shey Wiysonge, Abdu Abdullahi Adamu

**Affiliations:** 1 World Health Organization Regional Office for Africa, Brazzaville, Congo; 2 Cochrane South Africa, South African Medical Research Council, Cape Town, South Africa

**Keywords:** Immunization, implementation research, Africa

## Editorial

Immunization is a “best buy” health intervention with a return on investment as high as US$44 for every dollar spent [1]. Moreover, routine immunization is at the core of primary health care, and strengthening it is tightly linked with overall progress toward universal health coverage (UHC) [2]. The 2023 WHO-UNICEF Estimates of National Immunization Coverage (WUENIC) were an eye-opener for the WHO African Region as they highlighted that the region is still far off-track from achieving the Immunization Agenda 2030 (IA2030) impact goals [3]. Currently, efforts from Global Health Initiatives, notably Gavi, the Vaccine Alliance, complement government efforts to improve the availability of vaccines in low- and middle-income countries (LMICs) in the WHO African Region [4]. However, the ability of countries to deliver these vaccines equitably across communities remains a persistent challenge. This creates an immunization programme performance gap that undermines herd immunity and accentuates population susceptibility to vaccine-preventable diseases (VPD) outbreaks. It also worsens health and socioeconomic inequities and deters progress towards UHC.

It is estimated that the number of unvaccinated and under-vaccinated children in the African Region was 6.7 million and 9.9 million in 2023, respectively [3]. Most of these children are concentrated in 10 countries, with the Democratic Republic of Congo, Ethiopia, and Nigeria accounting for the highest numbers [3]. Insights into the complex interplay of supply and demand-side factors that drive immunization performance expose the significance of contextual variation between and within countries [5,6]. This is why vaccination efforts can be successful in one setting but fail in another [7]. Implementation research can help country immunization programmes better understand why vaccination efforts fail in specific settings, especially the feedback mechanisms in the interactions between multilevel factors, and how to use appropriate multicomponent and practical strategies tailored to address vicious loops or sustain virtuous loops [8].

Furthermore, implementation research has the necessary tools to catalyze a paradigm shift towards ‘precision immunization programming’ to reduce zero-dose and under-vaccinated children and sustain performance gains, by enhancing adaptation and resilience through adaptive systems learning [8]. Advancing adaptation and resilience in immunization systems, especially in African countries are particularly critical at this time as global health faces significant financial distress due to changing geopolitical landscape. There is already some interest in immunization learning agendas, but this is still inconsistent in the African region. Beyond agendas, there is a need for more substantial investments in transforming immunization programmes into active learning systems rooted in implementation research to strengthen local data use so that contextual information is rapidly and continually used to modify and improve approaches to vaccination efforts across diverse settings. Implementation research can be imagined as the convergence point between research and health programme delivery in real-world settings. It allows researchers to approach a systems problem through a policy lens, spurring methodological innovations that can address decision needs while emphasizing flexibility and timeliness compared to traditional research [9]. In the same way, implementation research also allows decision-makers to engage in research to ensure alignment with local needs and interests [9]. For this reason, purposeful implementation research to advance vaccination will need to be co-created, and its usefulness depends on the extent of collaborations between researchers, policymakers, Expanded Programme on Immunization (EPI) managers, frontline health workers, vaccination recipients, and other stakeholders involved in vaccination service delivery as obtainable within a particular country’s framework. Facilitating country-specific memoranda of understanding between stakeholders can enhance collaborations to accelerate progress towards transforming immunization programmes into learning systems. The value of implementation research in immunization is not limited to already existing vaccines. Implementation research can bridge the gap between new vaccines and national policies. Using the knowledge of implementation research, policymakers can identify and prioritize context-relevant implementation evidence needed for decisions regarding new vaccines and immunization-related technologies regardless of their stage in the development pipeline. This can fast-track the use of new vaccines in practice settings once they become available.

Institutionalizing implementation research in immunization programmes requires sustainable funding and commitment of local health authorities to attract trained implementation scientists and incentivize collaboration. While existing partners and global health initiatives can invest in implementation research as a key ingredient for strengthening learning systems in immunization programmes in the short term, the focus in the medium and long term should be on domestic resources. As immunization programmes break down siloes to maximize fiscal allocation from health and non-health sectors through a whole-of-government and whole-of-society approach, implementation research should be considered a critical cross-cutting area of investment for systems strengthening. In addition, countries must prioritize the systematic documentation of implementation strategies used to improve vaccination efforts to enable cross-context knowledge exchange to allow for replication in other places. A framework for Documenting and Reporting Implementation strategies of Vaccination Efforts (DRIVE), which was recently conceived, can support such efforts [10].

In conclusion, achieving globally set immunization programme performance targets in this era of funding shortages will require country-level stakeholders to embrace new ways of working that underscore rapid context-specific adaptation and system resilience. This can be enabled using implementation research. Therefore, the next few years provide an opportunity to embed and strengthen the use of implementation research in immunization programmes in Africa to promote localized problem-solving through learning. We call on governments, private sector entities, and global health initiatives to prioritize implementation research capacity strengthening in immunization programmes. To bridge the gap between aspiration and impact, we can make implementation research the heartbeat of immunization programmes; transforming knowledge into action, and challenges into breakthroughs, for every community, everywhere in Africa. Yes, we can!
